# Early Manifestations, Diagnostic Pathways, and Epilepsy in Juvenile-Onset Huntington Disease: A Three-Patient Case Series and Systematic Review

**DOI:** 10.3390/brainsci16080893

**Published:** 2026-08-21

**Authors:** Mirjana Perkovic Benedik, Tanja Loboda, Katarina Benedik Kafol, Jan Kafol, Neli Bizjak

**Affiliations:** 1 Department of Child, Adolescent and Developmental Neurology, University Children’s Hospital, University Medical Centre Ljubljana, 1000 Ljubljana, Slovenia; 2 Faculty of Medicine, University of Ljubljana, 1000 Ljubljana, Slovenia; 3 Faculty of Arts, University of Ljubljana, 1000 Ljubljana, Slovenia; 4 Department of Vascular Diseases, University Medical Centre Ljubljana, 1000 Ljubljana, Slovenia

**Keywords:** juvenile-onset Huntington disease, epilepsy, seizures, *HTT* CAG repeat expansion, basal ganglia, systematic review

## Abstract

**Highlights:**

**What are the main findings?**

- Juvenile-onset Huntington disease frequently presented with heterogeneous, non-choreic developmental, psychiatric, gait, speech, and movement abnormalities, with a median diagnostic delay of 4 years.
- Among published patients with ascertainable seizure status, epilepsy was reported in 41.4% and was descriptively more frequent in childhood-onset than adolescent-onset disease.

**What are the implications of the main findings?**

- Juvenile-onset Huntington disease should be considered when seizures or status epilepticus occur within a progressive multisystem neurological phenotype.
- HTT repeat-expansion testing should be considered despite absent family history, initially normal imaging, or non-diagnostic metabolic, gene-panel, or exome testing.

**Abstract:**

Background: Juvenile-onset Huntington disease (JoHD) is a rare form of Huntington disease characterized by symptom onset at or before 20 years of age. Early manifestations are often non-choreic and may be attributed to developmental, psychiatric, movement, metabolic, or epileptic disorders. We described three molecularly confirmed cases and examined early manifestations, diagnostic pathways, and epilepsy. Methods: We conducted a retrospective case series and a Preferred Reporting Items for Systematic Reviews and Meta-Analyses (PRISMA) 2020 systematic review of PubMed, Scopus, and Web of Science Core Collection through 5 July 2026. The strict patient-level synthesis required attributable onset at or before 20 years, patient-specific molecular confirmation of a pathogenic *HTT* repeat expansion, and extractable clinical data. Complementary aggregate or linked reports using closely aligned JoHD criteria were retained for context but excluded from patient-level calculations. Results: The cases included childhood-onset JoHD with drug-resistant epilepsy, adolescent-onset JoHD with progressive motor-cognitive decline and epilepsy in a known Huntington disease pedigree, and childhood-onset JoHD without available family history, in whom status epilepticus prompted renewed diagnostic evaluation. Ninety-three reports were included; of these, 81 contributed 228 unique patients and 12 provided complementary data. Early manifestations were heterogeneous and broadly consistent with previously described childhood-onset JoHD phenotypes. Diagnostic delay was extractable in 180/228 patients; among 172 with point estimates, the median was 4.0 years. Definite epilepsy was reported in 60/145 patients with ascertainable seizure status and was descriptively more frequent in childhood-onset (<10 years) than adolescent-onset (10–20 years) JoHD (49/84 [58.3%] vs. 11/57 [19.3%]). Conclusions: JoHD should be considered in children and adolescents with progressive multisystem neurological involvement, particularly when epilepsy occurs with developmental regression, gait or speech deterioration, pyramidal or extrapyramidal signs, basal-ganglia abnormalities, or a compatible family history.

## 1. Introduction

Huntington disease (HD) is an autosomal dominant neurodegenerative disorder caused by a cytosine-adenine-guanine (CAG) repeat expansion in the *HTT* gene [1]. Although rare, HD is a severe and ultimately fatal disease, characterized by progressive motor, cognitive, psychiatric, and functional decline. Recent meta-analytic data estimate the pooled global prevalence of HD at approximately 4.9 per 100,000 persons, with higher prevalence in populations of European ancestry [2]. The disease usually manifests in adult life, most often between 40 and 50 years of age, with median survival of approximately 15–18 years after clinical onset [3]. Juvenile-onset Huntington disease (JoHD), commonly defined as symptom onset at or before 20 years of age, represents only a small proportion of HD cases [4]. The earlier literature and meta-analytic data suggest that JoHD accounts for approximately 5% of HD cases, although reported estimates vary and more recent registry-based studies suggest that actively recognized JoHD cases may be even less frequent [5,6]. Compared with adult-onset HD, JoHD is associated with larger CAG repeat expansions, more frequent paternal transmission, delayed diagnosis, and a more rapidly progressive course [6,7]. Reported diagnostic delay should be interpreted cautiously, as JoHD onset may be assigned from early cognitive, behavioral, developmental, or motor changes, whereas adult-onset HD often uses motor-manifest onset; clinical recognition may also precede molecular confirmation [4,8].

At the molecular level, the expanded *HTT* gene produces mutant huntingtin, an abnormal form of a widely expressed protein involved in several cellular processes. Mutant huntingtin disrupts neuronal function through multiple mechanisms, including impaired transcription, mitochondrial dysfunction, disturbed proteostasis, synaptic dysfunction, neuroinflammation, and somatic CAG repeat instability [9,10]. Neurodegeneration is particularly prominent in the striatum, but cortical and other subcortical networks are also involved as the disease progresses [3,9]. Altered huntingtin function may disrupt brain development, including the formation and maturation of cortical and striatal circuits, potentially contributing to the early developmental and neurological phenotype of JoHD [11,12]. Adult-onset HD classically presents with chorea, psychiatric symptoms, and cognitive decline, whereas JoHD—particularly in younger children—is often non-choreic. Early manifestations may include developmental or learning difficulties, behavioral change, dysarthria, gait disturbance, rigidity, bradykinesia, dystonia, and seizures [7,13]. This atypical phenotype may resemble more common pediatric developmental, psychiatric, movement, metabolic, or epileptic disorders and thereby delay recognition.

Epilepsy is an important age-dependent feature of JoHD and is uncommon in adult-onset HD. In a multicenter cohort of 90 genetically confirmed patients with JoHD, Cloud et al. reported seizures in 38%, with greater frequency at younger ages of disease onset. Seizure phenotypes were heterogeneous, including generalized tonic-clonic, tonic, and myoclonus seizures and staring spells, and multiple seizure types could occur in the same patient [14]. Seizures may also occur at disease presentation; a previous systematic review reported seizures among presenting features in 26/285 (9.1%) JoHD cases overall and in 19/127 (15.0%) childhood-onset cases [13]. Subsequent reports have further highlighted severe epilepsy and progressive myoclonus epilepsy-like presentations in childhood-onset disease [15], as well as the difficulty of distinguishing epileptic myoclonus from cortical reflex myoclonus and other movement phenomena [16]. Nevertheless, evidence regarding electroencephalographic (EEG) characteristics, antiseizure treatment, and long-term epilepsy outcomes remains limited [14,15,16,17].

Treatment of HD and JoHD remains mainly symptomatic and supportive, as no approved disease-modifying therapy is currently available in routine clinical practice [18]. Management requires a multidisciplinary approach addressing motor, psychiatric, nutritional, communication, mobility, genetic counseling, and palliative care needs. In patients with JoHD and epilepsy, seizure recognition and appropriate antiseizure treatment represent additional clinical priorities.

Nance and colleagues described a characteristic childhood-onset HD profile comprising a family history of HD and two or more of declining school performance, seizures, oral-motor dysfunction, rigidity, and gait disturbance [19]. These established features provide an important diagnostic framework; the present study was not designed to identify new diagnostic symptoms or validate their predictive value, but to quantify early manifestations in molecularly confirmed JoHD and examine their relationship to diagnostic pathways and epilepsy.

Because JoHD is rare and clinically heterogeneous, evidence remains fragmented across cohorts, case series, and individual reports. We therefore combined three clinical cases with a systematic review focused on the earliest attributable manifestations and the pathway from symptom onset to molecular diagnosis. Secondary objectives were to characterize epilepsy and EEG findings and to summarize relevant genotype, neuroimaging, treatment, and outcome data.

## 2. Methods

### 2.1. Study Design and Case-Series Setting

We conducted a retrospective descriptive case series of three children with molecularly confirmed JoHD evaluated at the Department of Child, Adolescent and Developmental Neurology, University Children’s Hospital Ljubljana, Slovenia. These three patients represent all JoHD cases diagnosed in Slovenia between 2014 and 2026; no additional diagnosed cases were excluded. JoHD was defined as Huntington disease with symptom onset at or before 20 years of age and a pathogenic *HTT* CAG repeat expansion. The study followed the Declaration of Helsinki, and written informed consent for publication of anonymized clinical data was obtained from the patients’ parents or legal guardians.

### 2.2. Clinical Data Collection and Analysis

Medical records were reviewed from the earliest documented developmental or neurological concern to the latest available follow-up, encompassing the period 2014–2026. A standardized form captured developmental and family history, clinical manifestations, seizures, EEG, neuroimaging and genetic findings, treatment, functional course, and outcome. Age at molecular confirmation and *HTT* CAG repeat length were recorded for all three patients. Because of the descriptive design and small sample, findings were summarized narratively without inferential analysis.

### 2.3. Systematic Literature Review

The systematic review was conducted according to PRISMA 2020 [20]; the protocol was not prospectively registered. PubMed, Scopus, and Web of Science Core Collection were searched from inception to 5 July 2026 using database-adapted combinations of controlled vocabulary and free-text terms for Huntington disease and juvenile, childhood-onset, pediatric or paediatric, and Westphal phenotypes. Eligibility was limited to English-language reports, with no publication-date restriction. Unobtainable full texts were recorded as not retrieved. Citation searching comprised backward reference-list screening of included reports and relevant reviews to identify additional eligible publications. Complete search strategies and record counts are provided in Supplementary Table S1.

### 2.4. Eligibility and Evidence Sets

Reports were assigned to either a strict patient-level synthesis or a complementary evidence set. The strict synthesis included original case reports, case series, letters containing original patient data, and observational or registry studies describing individually identifiable patients with: (1) symptom onset attributable to Huntington disease at or before 20 years of age; (2) patient-specific molecular confirmation of an *HTT* CAG repeat expansion; and (3) extractable clinical data. Reports containing mixed-age populations contributed only when data for patients with onset at or before 20 years could be extracted separately.

Complementary reports were retained for narrative context when they provided additional information on patients or cohorts represented elsewhere or described aggregate molecularly confirmed JoHD cohorts from which eligible individual patient-level data could not be extracted. Reviews, editorials, books or book chapters, animal or laboratory studies, reports without molecular confirmation, conference abstracts without sufficient clinical information, and non-English reports were excluded.

### 2.5. Screening, Linkage, and Extraction

**Figure 1:**
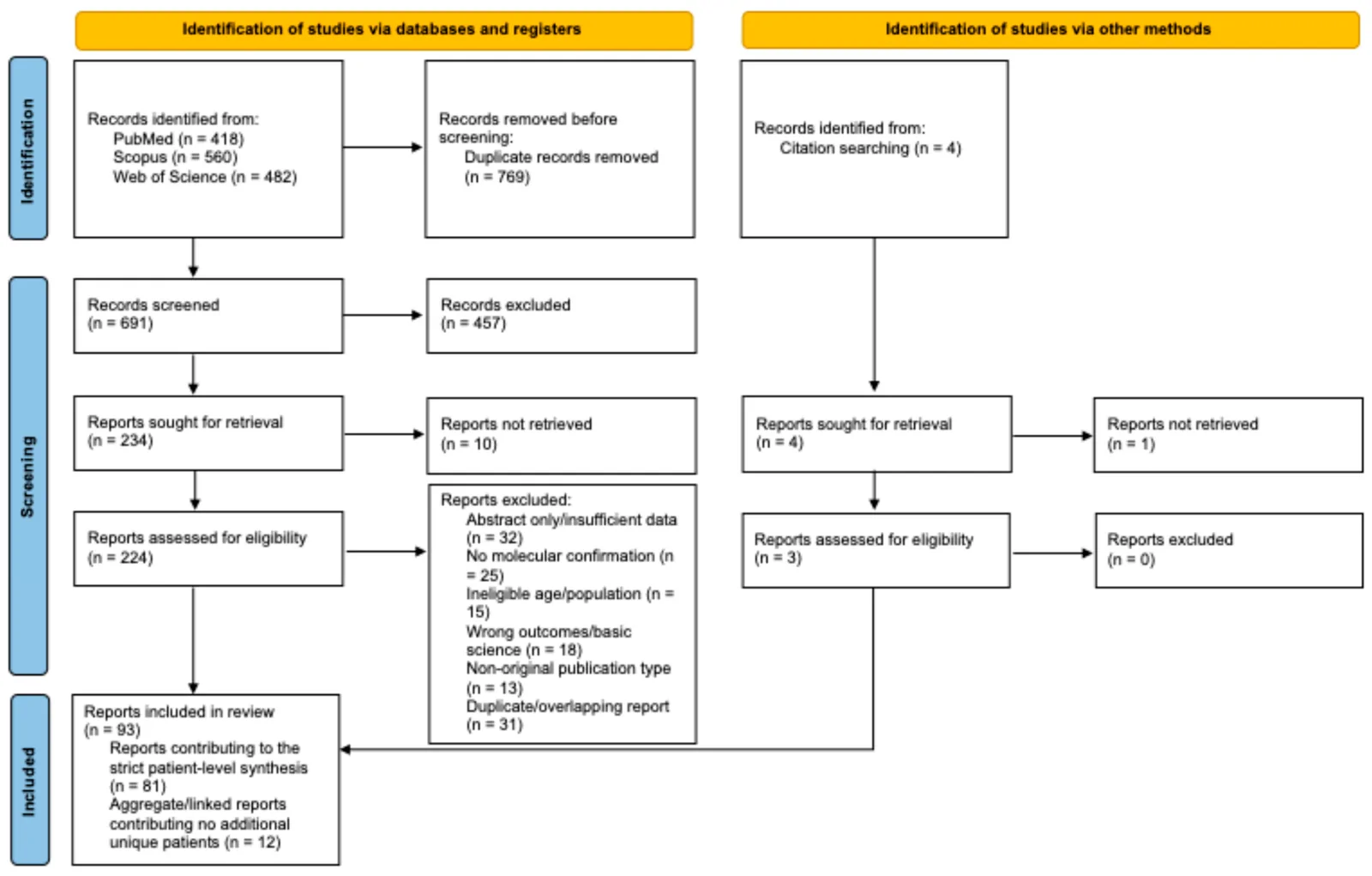
Preferred Reporting Items for Systematic Reviews and Meta-Analyses (PRISMA) 2020 flow diagram of study identification, screening, eligibility assessment, and inclusion [20]. Overall, 93 reports were included: 90 identified through database searching and three through citation searching. Eighty-one reports contributed 228 unique patients to the strict patient-level synthesis, whereas 12 complementary aggregate or linked reports provided contextual information without adding patient rows or contributing to patient-level estimates.

Records were deduplicated before screening. Reports describing the same patient or cohort were linked using bibliographic, institutional, pedigree, genetic, and distinctive clinical information. Each identifiable patient was counted once; follow-up reports were retained only when they contributed unique data. One review author screened records, assessed full texts, linked reports, and verified all eligibility decisions, exclusion reasons, report linkages, and extracted data against the source publications. ChatGPT 5.6 Sol (OpenAI) was used as an AI-assisted decision-support tool for preliminary screening, report linkage, provisional exclusion coding, and prepopulation of predefined extraction fields. Every AI-assisted output was manually verified against the corresponding source publication, no record was excluded solely on the basis of AI output, and all final eligibility decisions, report linkages, and extracted data were determined by the authors. The selection process is shown in Figure 1. Full bibliographic citations and study-level characteristics for all included reports are provided in Supplementary Tables S2 and S3 [6,7,8,15,16,17,21,22,23,24,25,26,27,28,29,30,31,32,33,34,35,36,37,38,39,40,41,42,43,44,45,46,47,48,49,50,51,52,53,54,55,56,57,58,59,60,61,62,63,64,65,66,67,68,69,70,71,72,73,74,75,76,77,78,79,80,81,82,83,84,85,86,87,88,89,90,91,92,93,94,95,96,97,98,99,100,101,102,103,104,105,106,107]. Details of excluded full-text reports are provided in Supplementary Table S4.

Extracted variables included publication characteristics, sex, transmitting lineage, expanded *HTT* CAG repeat length, earliest attributable manifestation and age at onset, motor onset, first seizure, molecular diagnosis, diagnostic delay, seizure semiology, status epilepticus, EEG and neuroimaging findings, treatment, functional course, and outcome.

Methodological quality was assessed by one reviewer using the Joanna Briggs Institute critical appraisal checklist appropriate to each study design [108]. No composite score was calculated; appraisal findings informed interpretation but were not used as the sole basis for exclusion. Detailed assessments are provided in Supplementary Table S5.

### 2.6. Outcome Definitions and Synthesis

Across both study components, age at onset was defined as the age of the earliest persistent manifestation subsequently considered attributable to JoHD; when exact timing was unavailable, it was estimated from the nearest documented history.

In the review, early manifestations were coded across seven non-mutually exclusive domains: (1) developmental delay or regression, cognitive or learning difficulties, or school decline; (2) behavioral or psychiatric manifestations; (3) gait imbalance, falls, or ataxia; (4) movement-disorder presentations, including dystonia, tremor, tics, chorea, or myoclonus; (5) speech, language, or oral-motor dysfunction; (6) rigidity, bradykinesia, or parkinsonism; and (7) epileptic seizures. A domain was counted as onset-present only when it was among the earliest attributable manifestations. Domain-specific denominators included only patients with ascertainable onset-domain status; missing or unclear reporting was not treated as absence.

Diagnostic delay was defined from the earliest persistent JoHD-attributable manifestation to molecular confirmation. A quantitative delay was calculated only when both time points could be assigned as exact ages or source-reported single-age estimates; ranges or imprecise descriptions were retained descriptively and were not converted into precise values. Explicitly approximate single values were also accepted for CAG repeat length and age at first seizure. Range-valued, interval-valued, inequality-based, or otherwise unclassifiable observations were excluded from point-estimate calculations; approximate or interval-valued diagnostic delays were summarized separately.

Childhood-onset JoHD was defined as onset before 10 years and adolescent-onset JoHD as onset from 10 to 20 years. Age-group assignment was based on the source-reported age of the earliest persistent JoHD-attributable manifestation and was independent of CAG-repeat length; repeat size was not used to infer age at onset. Age-stratified analyses included only patients assignable to one of these groups. Patients with unclassifiable onset age remained in the overall analysis, so subgroup totals did not necessarily equal the overall total.

Epilepsy was defined as recurrent unprovoked seizures or a first unprovoked seizure with documented clinical or EEG evidence of a high recurrence risk; for the review, a diagnosis of epilepsy in the source report also met the definition [109]. Patients entered the review epilepsy denominator only when clinical seizure status was explicitly ascertainable; source silence was treated as missing. Remote febrile seizures, isolated unprovoked seizures without documented high recurrence risk, acute symptomatic seizures, epileptiform EEG abnormalities without clinical seizures, and cortical reflex myoclonus without clinical epilepsy were excluded. Tremor or other abnormal movements without impaired awareness or an electrographic correlate were not classified as seizures. Status epilepticus was recorded when explicitly diagnosed or when the reported duration and clinical description met the operational International League Against Epilepsy definition for the relevant seizure type; emergency antiseizure treatment alone was insufficient [110].

Because the evidence consisted predominantly of case reports and small retrospective series with incomplete and heterogeneous reporting, no meta-analysis or formal comparative testing was performed. Patient-level summaries were calculated exclusively from the strict dataset using variable-specific denominators and are reported as counts, proportions, medians, and ranges. Descriptive analyses were performed using Microsoft Excel (Microsoft Corporation, Redmond, WA, USA), and the figure was generated in R (https://www.r-project.org/) (R Foundation for Statistical Computing, Vienna, Austria) using the ggplot2 package. The three index patients were analyzed separately from the systematic review and were not included in review-derived counts, which were restricted to patients identified from previously published reports.

## 3. Results

### 3.1. Case Series

The series comprised three children with genetically confirmed JoHD (Table 1). Patients 1 and 2 were siblings from a multigenerational paternal HD pedigree, whereas Patient 3 was adopted and had no available biological family history. The initial diagnostic evaluations occurred at ages 7–8 years in Patient 1, 13–14 years in Patient 2, and 6–9 years in Patient 3, reflecting different stages of disease evolution and genetic testing availability.

### 3.2. Patient 1

**Figure 2:**
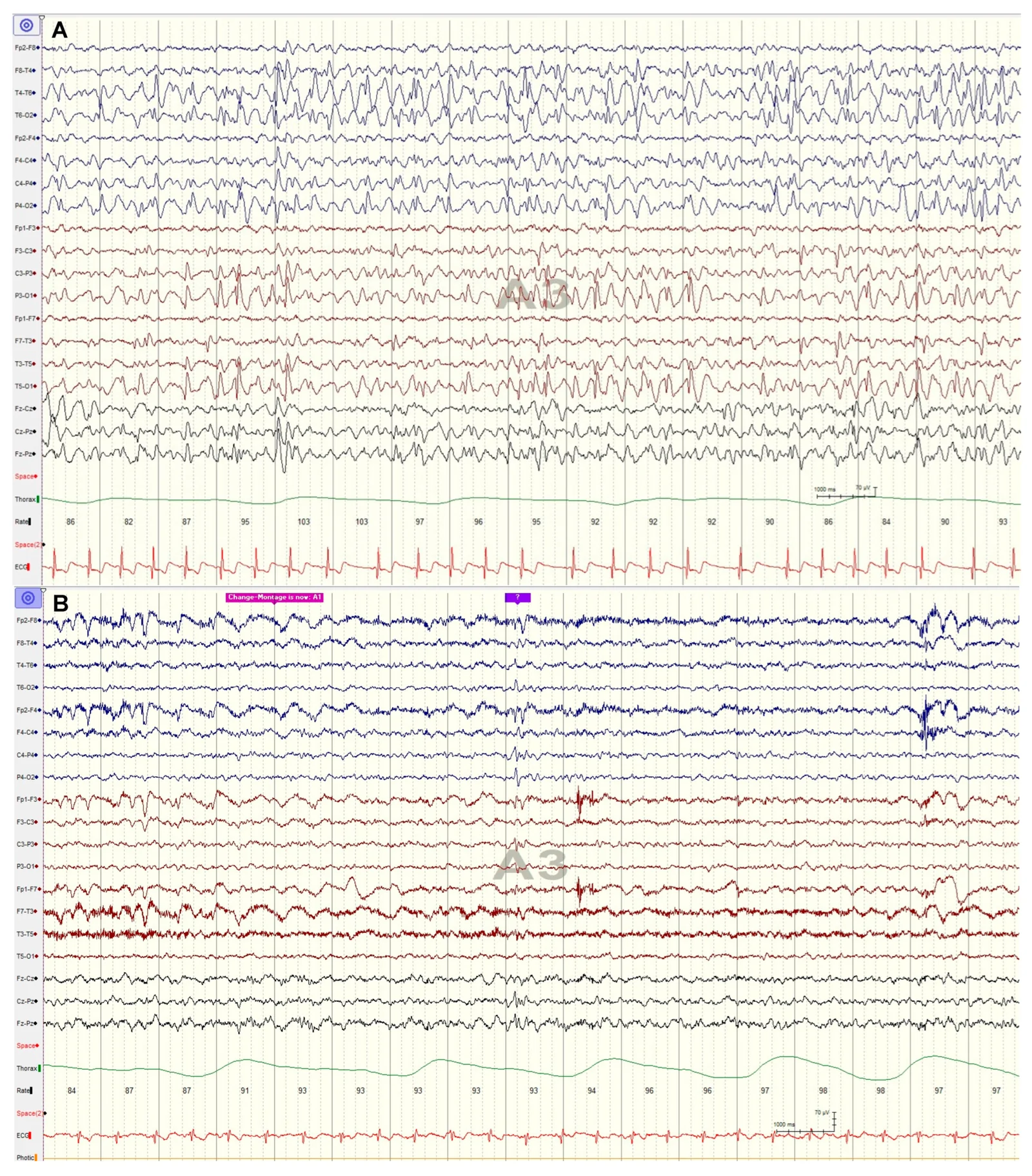
Representative electroencephalography (EEG) findings in Patients 1 and 2 with juvenile-onset Huntington disease. (**A**) Patient 1: EEG after the first seizure showing slow, disorganized background activity with very frequent, partly near-continuous focal epileptiform discharges over the left temporo-occipital and right temporoparietal-occipital regions. (**B**) Patient 2: EEG after the first seizure showing poorly modulated background activity with occasional sharp waves.

Patient 1 was a girl born after an uncomplicated term pregnancy. She walked shortly after 1 year of age and began speaking early, although her speech remained poorly articulated. From early childhood, she was noted to have longstanding motor coordination difficulties. Learning and attention difficulties later required transfer to an adapted school program. At 7 years, she suddenly lost balance during a school walk, fell without head trauma, became unresponsive, and developed a generalized tonic-clonic seizure lasting approximately 1 min. Examination documented axial hypotonia, intermittent limb hypertonia, impaired coordination, and a broad-based gait. EEG showed slow, disorganized background activity with very frequent, partly near-continuous independent epileptiform discharges over the left temporo-occipital and right temporoparietal-occipital regions, without a clinical correlate (Figure 2A), and valproate was initiated.

**Figure 3:**
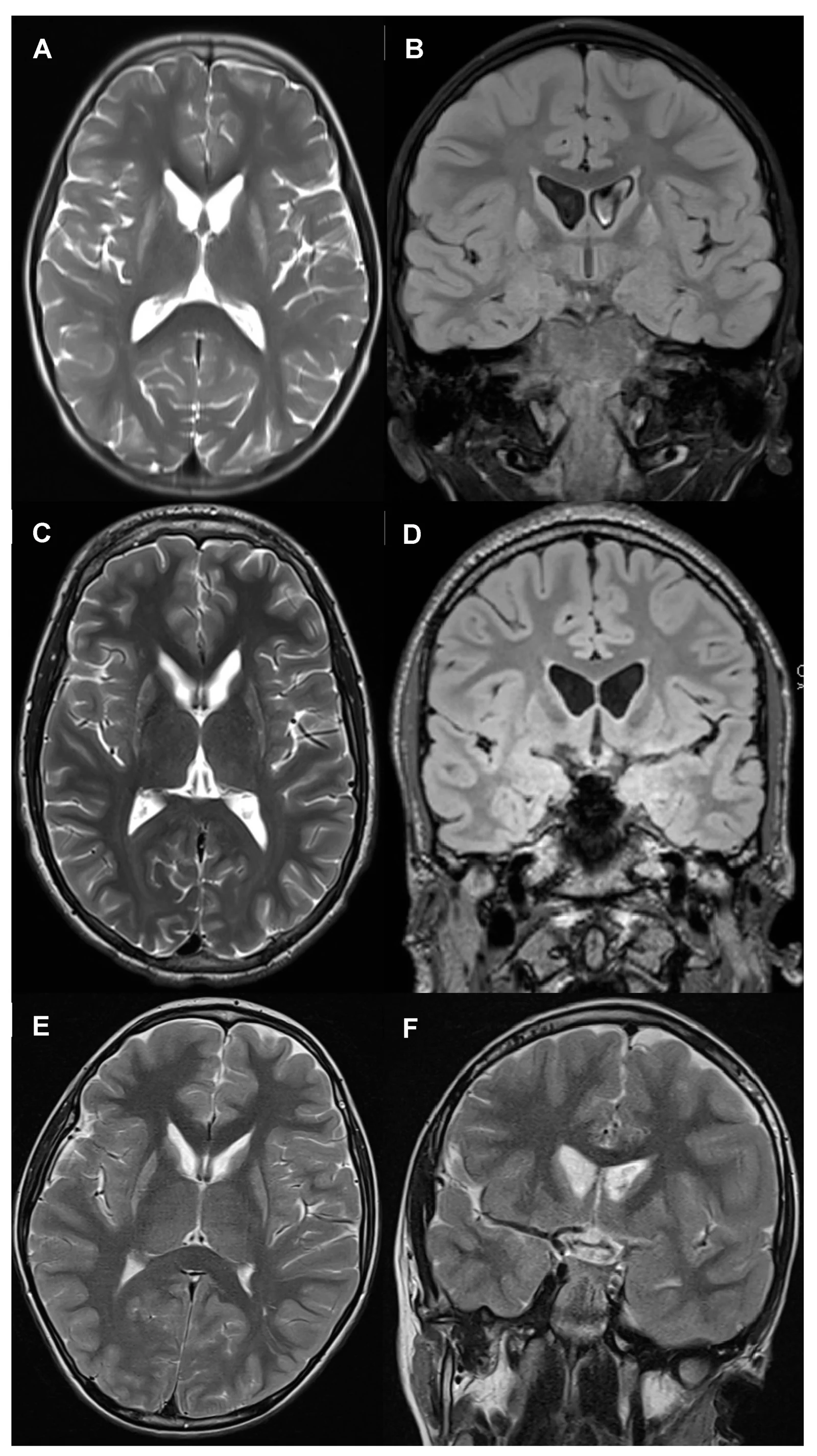
Brain MRI findings in the three patients with juvenile-onset Huntington disease. (**A**) Axial T2-weighted image showing atrophy of the lentiform nuclei in Patient 1. (**B**) Coronal fluid-attenuated inversion recovery (FLAIR) image showing atrophy of the caudate heads in Patient 1. (**C**) Axial T2-weighted image showing atrophy of the lentiform nuclei in Patient 2. (**D**) Coronal FLAIR image showing severe atrophy of the caudate heads in Patient 2. (**E**) Axial T2-weighted image showing increased signal intensity in the bilateral putamina and caudate nuclei in Patient 3. (**F**) Coronal T2-weighted image showing atrophy of the caudate nuclei in Patient 3.

Brain magnetic resonance imaging (MRI) the following month demonstrated mild caudate and putaminal atrophy, increased T2 signal in the striatum, and mild frontal-horn enlargement, initially interpreted as possible metabolic encephalopathy (Figure 3A,B). Extensive metabolic, cerebrospinal fluid, ophthalmologic, and chromosomal microarray investigations performed at age 7–8 years were non-diagnostic. Seizures recurred approximately every 6 weeks despite valproate, while serial EEG recordings showed multifocal and bilateral frontocentral epileptiform activity. During the following year, tremor, gait ataxia, myoclonus, rigidity, dysarthria, drooling, impaired coordination, and frequent falls progressed rapidly; levetiracetam and later clonazepam were added. A detailed pedigree then disclosed genetically confirmed Huntington disease in her father, paternal aunt, and paternal grandmother. Targeted *HTT* testing showed alleles with 25 and 106 CAG repeats, confirming JoHD. The epilepsy evolved into a drug-resistant progressive myoclonus epilepsy, with reports of up to 20 daily episodes and almost continuous central epileptiform activity. By 8.5 years, she had lost independent ambulation and required help with feeding and daily activities. Because of progressive dysphagia, weight loss, and difficulty taking medications, a percutaneous endoscopic gastrostomy was placed for enteral nutritional supplementation and medication administration. Despite symptomatic treatment with valproate, levetiracetam, clonazepam, levodopa/carbidopa, and rescue benzodiazepines, there was rapid progression of JoHD. She died at age 9 years outside the hospital; the cause of death was not documented in the available medical records.

### 3.3. Patient 2

Patient 2, the older brother of Patient 1, was born at term and had apparently normal early development, walking independently at 12 months. His medical history included myopia, convergent strabismus, and amblyopia. He also had nystagmus and had previously been followed for unexplained ataxia; however, the age at onset of the ataxia was not documented. At 13.5 years, worsening gait instability, whole-body tremor, and falls prompted neurological assessment. Because his sister had already been diagnosed with JoHD and his father had HD, genetic evaluation was recommended, but testing was not completed at that time. The tremor was initially most evident in unfamiliar situations, and an anxiety-related or functional component was considered. Examination nevertheless showed horizontal nystagmus, intention tremor, and an unsteady gait. EEG demonstrated mildly slowed and poorly modulated background activity without definite epileptiform discharges. Over the next year, the tremor became persistent and was accompanied by cognitive decline, slowed speech, and transfer to an adapted school program. At 14 years and 8 months, he became unresponsive while seated at school and developed a generalized tonic–clonic seizure characterized by generalized shaking and salivation. The seizure lasted approximately 3–4 min and was followed by fatigue and amnesia for the event.

During admission, examination revealed bradykinesia, dystonic hand posturing, limb rigidity, hyperreflexia, sustained ankle clonus, extensor plantar responses, dysmetria, impaired fine motor control, and a broad-based ataxic gait. EEG in wakefulness and after sleep deprivation showed diffuse slowing and intermittent generalized irregular spike-and-wave discharges with bifrontal predominance. A representative segment showing poorly modulated background activity with occasional sharp waves is shown in Figure 2B. MRI demonstrated pronounced bilateral caudate and putaminal atrophy with ex vacuo frontal-horn enlargement; magnetic resonance spectroscopy of the right striatum showed markedly increased myo-inositol at echo time (TE) 30 ms, consistent with gliosis, while no additional definite spectral abnormality was identified at TE 135 ms (Figure 3C,D). Given his affected father and his sister’s confirmed JoHD, targeted *HTT* analysis was performed and showed alleles of 17 ± 1 and 78 ± 3 CAG repeats. Valproate initially prevented further seizures, but motor and cognitive decline continued, with severe tremor, rigidity, dysarthria, word-finding difficulty, early contractures, and wheelchair dependence by 16 years. At that age, he developed clusters of focal-to-bilateral tonic-clonic seizures beginning with right-leg motor activity and head deviation. A discrepancy in the administered valproate dose was identified, and dose correction was followed by improved seizure control. Levodopa/carbidopa provided modest benefit for tremor and stiffness. He died at age 20 years and 3 months.

### 3.4. Patient 3

Patient 3 was adopted into Slovenia at 1 year of age; biological family history and perinatal details were therefore unavailable. He walked at approximately 12 months. Early concerns included poorly intelligible speech, sensory sensitivities, short attention span, and possible autistic features, although he remained sociable and largely independent. At 5–6 years, caregivers noted progressive gait instability, frequent falls, reduced gross and fine motor performance, fatigue, dysarthria, and occasional choking. Examination showed a variably ataxic gait with toe walking and foot dragging, impaired balance, poor heel walking, slow and inaccurate coordination, and fine motor difficulties. Routine laboratory testing and muscle enzymes were normal; EEG was normal for age; MRI was initially reported as normal apart from mild sinusitis; and ophthalmologic assessment, electromyography, metabolic screening, and a 206-gene metabolic panel were non-diagnostic. Subsequent next-generation sequencing testing identified heterozygous variants in *GLB1* and *SUMF1* that were considered non-explanatory, while chromosomal microarray analysis identified a 7q31.31 duplication classified as a variant of uncertain significance. Because some motor improvement was observed, post-infectious, developmental, and autism-spectrum explanations remained under consideration.

At 9 years, he presented with his first documented seizure, a focal-to-bilateral tonic-clonic status epilepticus, with leftward head and eye deviation, tonic extension, and bilateral shaking, lasting approximately 30–60 min. The seizure persisted despite initial diazepam and terminated after additional intravenous benzodiazepines and levetiracetam. Head computed tomography (CT) was normal. After recovery, examination showed dysarthria, reduced tongue mobility, slowed fine motor movements, generalized hypertonia, marked hyperreflexia, bilateral extensor plantar responses, impaired balance, and a poorly coordinated gait. Video-EEG two days after status epilepticus showed a symmetric, appropriately organized background without postictal slowing or convincing interictal epileptiform discharges; a single questionable right-posterior abnormality was noted. The recording followed acute treatment with benzodiazepines and levetiracetam, which may have suppressed interictal epileptiform activity; no subsequent EEG remote from antiseizure medication was available. No maintenance antiseizure medication was initiated at discharge; buccal midazolam was prescribed as rescue treatment. No seizure recurrence was documented before discharge. The combination of longstanding motor and speech abnormalities, pyramidal and extrapyramidal signs, and status epilepticus prompted renewed evaluation for neurodegeneration. Subsequent brain MRI demonstrated bilateral volume loss and T2 hyperintensity involving the caudate nuclei and putamina, with associated moderate ex vacuo enlargement of the frontal horns of the lateral ventricles (Figure 3E,F). Multivoxel magnetic resonance spectroscopy of the basal ganglia (TE 30 ms) showed moderately increased myo-inositol, mildly decreased N-acetylaspartate and choline, and an increased glutamate/glutamine peak. These findings were suggestive of JoHD. Whole-exome sequencing had been initiated during the diagnostic evaluation of his unexplained progressive neurodevelopmental phenotype. Subsequent analysis of the existing exome-sequencing data using ExpansionHunter (Illumina, San Diego, CA, USA) indicated a heterozygous HTT CAG-repeat expansion [111]. Although ExpansionHunter was primarily developed for analysis of PCR-free whole-genome sequencing data, the diagnostic report documented its application to the exome dataset; further details regarding this bioinformatic analysis were not available. The finding was subsequently confirmed by an independent molecular test (internal laboratory order 73195/HD), demonstrating an expanded HTT allele of 90 ± 1 CAG repeats. The available report did not specify the technical method used for this confirmatory assay. Because the patient was symptomatic, this constituted diagnostic rather than predictive genetic testing. Because the patient was adopted, the parental origin of the expansion could not be determined. At the last documented follow-up, aged 9 years and 3 months, he remained ambulatory but had persistent dysarthria, hypertonia, hyperreflexia, and impaired coordination.

### 3.5. Systematic Review

#### Literature Synthesis

Ninety-three reports were included: 90 identified through database searching and three through citation searching. Eighty-one reports contributed 228 unique molecularly confirmed patients to the strict patient-level synthesis, whereas 12 complementary aggregate or linked reports provided contextual information without adding patient rows or contributing to patient-level estimates. Study-level characteristics are presented in Supplementary Tables S2 and S3. Age at onset could be assigned to a broad stratum in 221 patients: 106 (48.0%) had childhood-onset JoHD (<10 years) and 115 (52.0%) had adolescent-onset JoHD (10–20 years); seven patients remained unclassifiable. Among 195 patients with a reported or inferable transmitting lineage, 158 (81.0%) had paternal inheritance. Expanded-allele CAG-repeat information was available for 212/228 patients (93.0%). Among 202 patients with a single numerical or explicitly approximate point estimate, the median was 68 repeats (range, 40–316); reported interval-valued expansions extended to 350 repeats.

**Figure 4:**
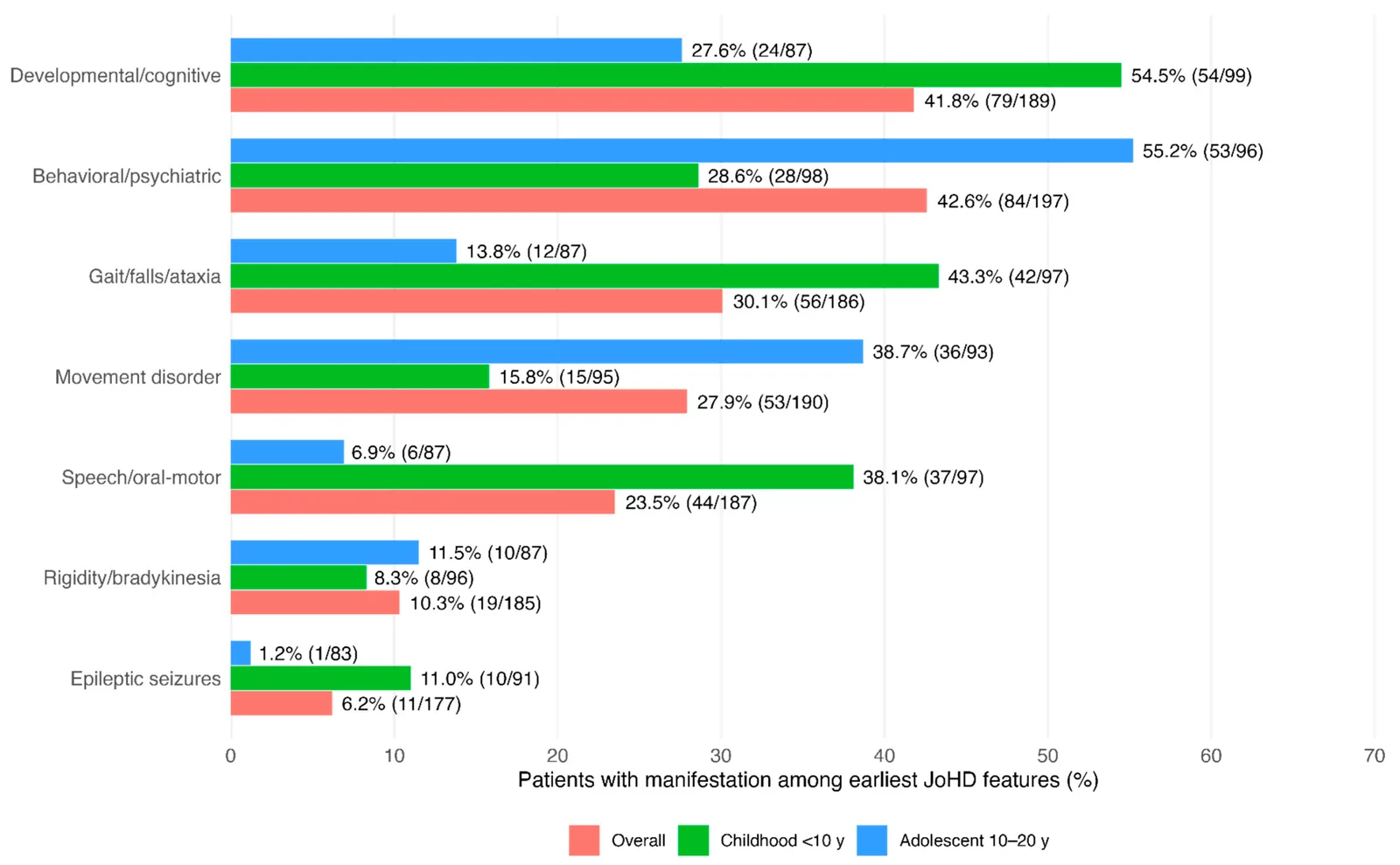
Early manifestations in juvenile-onset Huntington disease according to age at disease onset. Bars show the proportions of patients in the strict patient-level synthesis in whom each clinical domain was present among the earliest manifestations attributable to juvenile-onset Huntington disease. Overall estimates are shown together with estimates for childhood-onset disease, defined as onset before 10 years, and adolescent-onset disease, defined as onset from 10 to 20 years. Clinical domains were non-mutually exclusive, and patients could contribute to more than one domain. Percentages were calculated using variable-specific denominators that included only patients with ascertainable onset-domain status. Patients with unclassifiable onset age were included in overall estimates when applicable but excluded from age-stratified comparisons. Complementary reports did not contribute to the calculations. Abbreviations: JoHD, juvenile-onset Huntington disease.

The early phenotype was heterogeneous and frequently non-choreic. Developmental delay or regression, cognitive or learning difficulties, or school decline were reported among the earliest attributable manifestations in 79/189 (41.8%) patients, followed by behavioral or psychiatric manifestations in 84/197 (42.6%), gait imbalance, falls, or ataxia in 56/186 (30.1%), movement-disorder presentations in 53/190 (27.9%), speech, language, or oral-motor dysfunction in 44/187 (23.5%), rigidity or bradykinesia in 19/185 (10.3%), and epileptic seizures in 11/177 (6.2%). Descriptively, childhood-onset disease more often involved developmental or cognitive, gait, speech or oral-motor, and seizure-related manifestations, whereas behavioral or psychiatric and movement-disorder presentations were more common in adolescent-onset disease (Figure 4) [8,41,65,75,78,100,101].

### 3.6. Diagnostic Pathways and Delay

Diagnostic delay was ascertainable in 180/228 (78.9%) patients. Among 172 patients with exact or source-derived point estimates, the median interval between the earliest attributable manifestation and molecular diagnosis was 4.0 years (range, 0–26); eight additional patients had approximate or interval-valued delays. Reported alternative attributions and diagnostic pathways included attention-deficit/hyperactivity disorder, autism-spectrum disorder, learning disability, depression, psychosis, substance use, eating disorders, Tourette syndrome, essential tremor, cerebral palsy, Wilson disease, leukodystrophy, mitochondrial disease, and other inherited neurodegenerative disorders before *HTT* testing was pursued. Family history shortened the diagnostic route when it was known and available but was sometimes absent, undisclosed, unavailable because of adoption or parental separation, or initially uninformative when a transmitting parent had not yet been clinically recognized as affected. A child-before-parent diagnostic sequence was explicitly documented in one patient; parental diagnostic timing was otherwise too inconsistently reported to estimate its frequency across the cohort. The practical diagnostic signal was therefore the combination of progression, multisystem involvement, and pedigree context rather than any single presenting symptom [8,40,58,65,69,75,78,100,101,102,106].

### 3.7. Epilepsy and Neurophysiology

Epilepsy-related findings were analyzed separately from other paroxysmal or neurophysiological phenomena (Table 2). Among 145 patients whose clinical seizure status was explicitly ascertainable, definite epilepsy was reported in 60 (41.4%). Among the 85 patients not classified as having definite epilepsy, 14 had prespecified exclusionary phenomena: remote febrile seizures (*n* = 2), isolated seizures without documented recurrence or high recurrence risk (*n* = 2), acute symptomatic postoperative seizures (*n* = 2), epileptiform EEG abnormalities without clinical seizures (*n* = 4), and cortical or cortical-reflex myoclonus without clinical epilepsy (*n* = 4). Epilepsy was descriptively more frequent in childhood-onset disease, affecting 49/84 (58.3%) patients with onset before 10 years, compared with 11/57 (19.3%) patients with onset at 10–20 years. Epileptic seizures were among the earliest attributable manifestations in 11/177 (6.2%) patients with ascertainable onset-domain information. Age at first seizure was available as an exact or explicitly source-reported approximate single-age estimate for 40/60 patients with definite epilepsy, with a median of 6.5 years (range, 2–20). Status epilepticus was identified in 5/60 patients with epilepsy (8.3%).

EEG and neurophysiological findings were variably reported and included background slowing, epileptiform discharges, generalized polyspikes, and cortical-myoclonus physiology; aggregate or incompletely mapped reporting precluded calculation of a valid patient-level proportion. Several reports illustrated the electroclinical difficulty of distinguishing epileptic seizures or epileptic myoclonus from dystonia, chorea, stereotypies, non-epileptic myoclonus, or cortical reflex myoclonus, supporting careful electroclinical classification rather than assuming that all paroxysmal events represent epilepsy [7,15,16,17,43,85,92].

### 3.8. Neuroimaging, Genetics, Treatment, and Outcome

Neuroimaging or neuropathology findings were individually available for 121/228 (53.1%) patients. Striatal or broader basal-ganglia abnormalities were commonly described, although aggregate and incompletely mapped reporting precluded calculation of a reliable patient-level positivity proportion. Early imaging could nevertheless be normal, and very-high-repeat JoHD cases sometimes showed prominent cerebellar, diffuse cortical, or metabolic changes in addition to striatal involvement. Several reports also underscored that non-diagnostic exome sequencing, broad metabolic testing, or even a false-negative standard polymerase chain reaction assay does not exclude JoHD when the phenotype is progressive and repeat-expansion testing has not been performed. Treatment evidence remained descriptive and symptom-directed. Vesicular monoamine transporter 2 (VMAT2) inhibitors were reported in 14 patients (tetrabenazine in 13 and deutetrabenazine in one), including five childhood-onset and eight adolescent-onset cases; one patient had unclassifiable onset age. They were used for chorea or other hyperkinetic movements, dystonia, or mixed involuntary-movement phenotypes. These case-based reports do not establish the efficacy or safety of VMAT2 inhibitors in children with JoHD. Other reported treatments included antiseizure medications, levodopa, baclofen, clonazepam, antipsychotics, botulinum toxin, gastrostomy, deep-brain stimulation, intrathecal baclofen, and selected neurophysiology-guided therapies, but no disease-modifying effect could be inferred from the available descriptive evidence. Outcomes were severe in childhood-onset and high-repeat cases, with loss of ambulation, dysphagia, gastrostomy, respiratory complications, institutional care, and early death repeatedly documented [7,15,41,95,99,103,104,107].

## 4. Discussion

In this three-patient case series and systematic review, JoHD was characterized by heterogeneous, frequently non-choreic manifestations and delayed diagnostic recognition. Early symptoms commonly entered diagnostic pathways as more prevalent pediatric conditions, including developmental or learning difficulties, autism-spectrum features, psychiatric disorders, tremor, ataxia, epilepsy, metabolic disease, or other neurodegenerative disorders [13,78,101].

The three cases illustrate variations within a shared clinical pattern. Patient 1 had severe childhood-onset disease with drug-resistant epilepsy and rapid progression; Patient 2 had a slower adolescent-onset course in a known HD pedigree; and Patient 3 had no available biological family history, with status epilepticus prompting renewed diagnostic evaluation. The cases therefore differed mainly in age at onset, family-history availability, epilepsy severity, disease tempo, and diagnostic pathway.

CAG repeat length, age at onset, epilepsy, and family history influenced the diagnostic pathway without determining a uniform phenotype. Patient 1, with 106 repeats, resembled the highly expanded childhood-onset subgroup described by Fusilli et al., in which developmental impairment, gait abnormalities, seizures, rapid progression, and shorter survival clustered in patients with expansions above approximately 80 repeats [7]. Her brother, with 78 repeats, had a less fulminant course, supporting repeat length as an important but incomplete determinant of severity. Affected siblings with JoHD have been reported previously, and substantial intrafamilial variation in expanded CAG-repeat length can occur, including three siblings with 93, 100, and 120 repeats and two brothers with approximately 98 and 250–350 repeats [44,81]. Thus, the 106-versus-78-repeat difference in our siblings is notable but not unprecedented. In Patient 3, the absence of known family history shifted attention toward developmental, post-infectious, metabolic, and autism-spectrum explanations. Across the cases, the most informative signal was therefore progression across several domains rather than any isolated manifestation.

Previous reviews and cohort studies similarly showed that JoHD frequently presents with non-choreic behavioral, cognitive, speech, gait, parkinsonian, or epileptic features [13]. Highly expanded childhood-onset disease appears particularly associated with developmental disturbance, epilepsy, and rapid progression, consistent with combined neurodevelopmental and neurodegenerative mechanisms [7,11]. Registry studies have also demonstrated that diagnosis is often delayed until functional impairment is already established [6,101]. The present review extends this work by reconstructing the interval from the earliest attributable manifestation to molecular diagnosis in individually confirmed patients and by examining epilepsy quantitatively while synthesizing EEG and neurophysiological findings qualitatively. Of the 93 reports included in the present review, 44 were published after the May 2017 search cutoff of the previous systematic review by Cronin et al.; 37 contributed patient-level data and seven provided complementary contextual information [13].

The epilepsy findings are consistent with and extend previous observations. Cloud et al. reported seizures in 38% of genetically confirmed JoHD patients and demonstrated associations with younger age at onset and larger CAG expansions [14]. In our strict synthesis, definite epilepsy was identified in 41.4% of patients with explicitly ascertainable seizure status and was descriptively more frequent in childhood-onset than adolescent-onset disease (58.3% vs. 19.3%). These analyses capture different aspects of the phenotype: definite epilepsy reflects whether epilepsy occurred at any point in the reported disease course, whereas seizures among the earliest attributable manifestations indicate whether epilepsy contributed to the initial clinical presentation. Direct comparison is limited because reports silent on seizure status were excluded from our denominator and published cases are vulnerable to selection and publication bias. Seizures were among the earliest attributable manifestations in 6.2% of patients overall and 11.0% of those with childhood-onset JoHD, suggesting that epilepsy more often emerged within a broader trajectory of developmental, motor, speech, or cognitive deterioration than as an isolated diagnostic clue. These estimates are somewhat lower than the approximately 15% reported at presentation in childhood-onset cases in the previous systematic review, potentially reflecting differences in eligibility criteria, age-group definitions, and handling of incompletely reported onset features [13]. Status epilepticus was documented in 5/60 (8.3%) patients with epilepsy, indicating that prolonged and potentially severe seizure presentations form part of the JoHD epilepsy spectrum, particularly in selected childhood-onset cases.

A first seizure in an otherwise normally developing child should not automatically prompt *HTT* testing. Suspicion should increase when seizures or status epilepticus occur with progressive gait impairment, dysarthria, cognitive regression, dystonia-parkinsonism, pyramidal signs, myoclonus, basal-ganglia abnormalities, or a compatible family history. Careful electroclinical interpretation is therefore essential, because epileptiform EEG abnormalities do not establish epilepsy in the absence of compatible clinical events, while paroxysmal manifestations such as cortical reflex myoclonus, dystonia, chorea, tremor, tics, or stereotypies may mimic epileptic seizures. Misclassification may lead either to unnecessary antiseizure treatment or to failure to recognize a treatable contributor to morbidity [14,17,107].

Diagnostic testing should be interpreted in the context of the clinical trajectory. Although striatal or broader basal-ganglia abnormalities were frequently reported, early MRI could be normal or nonspecific, as in Patient 3, while very-high-repeat disease could produce broader cerebellar, cortical, or metabolic abnormalities [7,95,104]. Likewise, non-diagnostic metabolic investigations, gene panels, exome sequencing, or variants of uncertain significance do not exclude JoHD when repeat-expansion analysis has not been performed. *HTT* repeat-expansion testing should therefore remain in the diagnostic framework when progressive neurodevelopmental, motor, speech, cognitive, epileptic, imaging, and familial features converge.

Treatment evidence remained descriptive and symptom-directed, and no disease-modifying effect could be inferred [18]. Nevertheless, establishing the diagnosis remains clinically important because it can reduce repeated non-diagnostic investigations, clarify prognosis, guide seizure and symptom management, support educational and disability planning, enable genetic counseling, and facilitate anticipatory care for dysphagia, nutrition, mobility, respiratory complications, and end-of-life needs.

Several limitations should be acknowledged. The retrospective, single-center case series included only three patients and cannot estimate disease frequency, risk factors, or treatment effectiveness. The systematic review was based predominantly on case reports and small retrospective series and was therefore vulnerable to publication bias favoring severe, unusual, very-early-onset, large-CAG-expansion, or epilepsy-associated cases. Some complementary aggregate or linked reports did not permit extraction of individually identifiable eligible patients or substantially overlapped with patients represented in the strict dataset; these reports therefore contributed only to narrative context and not to patient-level estimates.

Reporting was incomplete and definitions of onset, motor onset, epilepsy, and diagnostic delay varied across reports. Many early manifestations and their timing were reconstructed retrospectively, making diagnostic-delay estimates susceptible to recall and attribution bias. Source silence was treated as missing rather than absence, requiring variable-specific denominators. Restriction to English-language publications may also have excluded relevant cases. Screening, report linkage, and extraction were performed by one review author with AI-assisted decision support, although all final decisions and data were verified against the source publications. Although the overall patient-level sample was relatively large, substantial missingness, heterogeneous reporting, and frequent use of approximate or interval-valued data reduced the number of patients with jointly available variables and precluded reliable multivariable modeling. Clinical and methodological heterogeneity precluded meta-analysis. The reported proportions and diagnostic-delay estimates should therefore be interpreted as a descriptive synthesis of published cases with extractable data rather than as population prevalence estimates or precise comparative measures of risk.

## 5. Conclusions

This study provides a patient-level synthesis of diagnostic delay and epilepsy across the JoHD spectrum and complements these findings with three clinically detailed cases. Epilepsy was descriptively more frequent in childhood-onset than adolescent-onset JoHD, while seizures were less common among the earliest manifestations and usually occurred within a broader progressive neurological phenotype. These findings support consideration of JoHD when seizures or status epilepticus accompany progressive multisystem neurological abnormalities, even when family history is unavailable or initial investigations are non-diagnostic.

## Figures and Tables

**Table 1:** Comparative clinical, epileptic, neurophysiological, neuroimaging, genetic, and outcome characteristics of the three patients with juvenile-onset Huntington disease.

Characteristic	Patient 1	Patient 2	Patient 3
Sex	Female	Male	Male
Relationship and family history	Siblings from a multigenerational paternal HD pedigree. Their father, paternal aunt, and paternal grandmother had genetically confirmed Huntington disease; the family history was recognized during the diagnostic work-up.		Adopted; biological family history unavailable.
Earliest attributable manifestations	Poorly articulated speech and longstanding motor coordination difficulties from early childhood, followed by learning and attention difficulties; first seizure at age 7 years.	Unexplained ataxia of undocumented onset; by 13.5 years, progressive gait instability, whole-body tremor, and falls were evident, followed by cognitive decline, slowed speech, and bradykinesia.	Speech and attention difficulties, followed by progressive gait instability, recurrent falls, impaired fine and gross motor function, and dysarthria.
Age at earliest attributable manifestation	Early childhood; exact age not documented.	Age at onset of ataxia undocumented; progressive gait instability, tremor, and falls documented by age 13.5 years.	Approximately 5–6 years.
Age at first seizure	7 years	14 years 8 months	9 years
Initial seizure phenotype	Generalized tonic–clonic seizure lasting approximately 1 min.	Generalized tonic–clonic seizure lasting approximately 3–4 min, with salivation, postictal fatigue, and amnesia for the event.	Focal-to-bilateral tonic–clonic status epilepticus, with head and eye deviation, tonic extension, and bilateral shaking, lasting approximately 30–60 min.
Seizure or epilepsy course	Recurrent drug-resistant epilepsy with progressive myoclonus epilepsy-like features and reports of up to 20 daily events.	Initially controlled epilepsy; later recurrent focal-to-bilateral tonic-clonic seizure clusters.	Single documented focal-to-bilateral tonic-clonic status epilepticus; no further seizures documented before discharge and no epilepsy diagnosis established.
EEG findings	Slowed and disorganized background with multifocal, frequently near-continuous epileptiform discharges.	Diffuse background slowing with generalized irregular spike-and-wave discharges; later recordings showed a left frontocentral epileptiform focus during drowsiness and sleep.	EEG 2 days after status: organized symmetric background; no postictal slowing or convincing epileptiform discharges; obtained after acute ASM treatment.
Brain MRI findings	MRI at age 7 years showed early caudate and putaminal atrophy with T2 striatal signal abnormalities, initially interpreted as possible metabolic encephalopathy.	MRI at age 14 years showed pronounced bilateral caudate and putaminal atrophy with ex vacuo enlargement of the frontal horns; magnetic resonance spectroscopy showed evidence of striatal gliosis.	MRI at age 6 years was initially reported as normal; at age 9 years, imaging showed bilateral caudate and putaminal volume loss and T2 hyperintensity with moderate ex vacuo frontal-horn enlargement; magnetic resonance spectroscopy showed metabolic abnormalities compatible with gliosis/neurodegeneration.
*HTT* CAG repeat lengths	25/106	17 ± 1/78 ± 3	Expanded allele: 90 ± 1 CAG repeats; non-expanded allele not reported
Age at molecular diagnosis	8 years 5 months	14 years 9 months	9 years 3 months
Estimated diagnostic delay	Several years; the exact interval could not be determined because the early-childhood manifestations were not dated precisely.	At least approximately 1 year from clearly documented progression; the true interval may have been longer because the onset of ataxia was undocumented.	Approximately 3–4 years from progressive gait and speech abnormalities to molecular confirmation.
Antiseizure medications	Valproate, levetiracetam, clonazepam; rescue benzodiazepines.	Valproate; rescue buccal midazolam.	Acute diazepam, lorazepam, and intravenous levetiracetam for status epilepticus; buccal midazolam prescribed for rescue; no maintenance ASM at discharge.
Other symptomatic/supportive management	Levodopa/carbidopa for motor symptoms; PEG placement for progressive feeding difficulties and weight loss, with enteral nutritional supplementation.	Levodopa/carbidopa.	Supportive multidisciplinary management; no specific chronic pharmacologic treatment reported.
Clinical course and outcome	Rapid motor and epileptic deterioration, with loss of ambulation, dysphagia, gastrostomy placement, and death at age 9 years.	Progressive motor and cognitive decline, with severe functional impairment and wheelchair dependence by age 16 years; he died at age 20 years and 3 months.	At discharge, he remained ambulatory but had persistent dysarthria, impaired coordination, hypertonia, hyperreflexia, and extensor plantar responses.
Role of epilepsy in the diagnostic pathway	First seizure at age 7 prompted specialist neurological evaluation of previously present developmental and motor abnormalities; JoHD was confirmed in 2016 after subsequent progression and pedigree clarification.	Known JoHD in his sister and HD in his father prompted genetic referral in 2018, but testing was not completed; his first seizure in 2019 prompted hospitalization and definitive *HTT* testing.	Status epilepticus prompted renewed consideration of a neurodegenerative disorder after several years of inconclusive investigations.

Patients 1 and 2 were siblings; Patient 3 was adopted and had no available biological family history. Ages at onset and diagnostic delays were estimated retrospectively when exact dates were unavailable. For Patients 1 and 2, *HTT* CAG repeat lengths are presented as shorter allele/expanded allele; for Patient 3, only the expanded allele was available. Abbreviations: ASM, antiseizure medication; CAG, cytosine-adenine-guanine; EEG, electroencephalography; HD, Huntington disease; *HTT*, huntingtin gene; JoHD, juvenile-onset Huntington disease; MRI, magnetic resonance imaging; PEG, percutaneous endoscopic gastrostomy.

**Table 2:** Epilepsy-related findings in juvenile-onset Huntington disease.

Epilepsy-Related Domain	Findings in the Strict Synthesis
Definite epilepsy by JoHD onset group	Epilepsy was documented in 60/145 (41.4%) patients with ascertainable seizure status: 49/84 (58.3%) with childhood-onset and 11/57 (19.3%) with adolescent-onset JoHD.
Seizures as an early manifestation	Seizures were among the earliest attributable manifestations in 11/177 (6.2%) patients. Among 40/60 patients with an available point estimate, the median age at first seizure was 6.5 years (range, 2–20).
Severe or difficult-to-control epilepsy	Status epilepticus occurred in 5/60 (8.3%) patients with epilepsy. Drug-resistant or difficult-to-control epilepsy was described in several childhood-onset cases.
Epileptic myoclonus/progressive myoclonus epilepsy–like presentations	Reported in selected childhood-onset cases, particularly in association with large CAG expansions.
EEG and neurophysiology	Findings included background slowing, epileptiform discharges, generalized polyspikes, and cortical-myoclonus physiology; heterogeneous reporting precluded a valid patient-level proportion.

Epilepsy comprised recurrent unprovoked seizures, a source diagnosis of epilepsy, or a first unprovoked seizure with documented high recurrence risk. Febrile or acute symptomatic seizures, isolated unprovoked seizures without high recurrence risk, EEG abnormalities without clinical seizures, and cortical reflex myoclonus without epilepsy were excluded. Source silence was treated as missing. Childhood-onset JoHD was defined as onset before 10 years and adolescent-onset JoHD as onset at 10–20 years.

## Data Availability

The original data supporting the findings of this study are available in a publicly accessible repository. The dataset has been deposited in the Open Science Framework (OSF) at the following link: [https://osf.io/mq5b8/overview?view_only=c1a190ebc24a45d5b6a0da86c1669c48] accessed on 23 July 2026.

## Supplementary Materials

The following supporting information can be downloaded at: https://www.mdpi.com/article/10.3390/brainsci16080893/s1, Table S1: Search strategies and database record counts; Table S2: Included reports and evidence tier; Table S3: Extraction for 228 unique patients; Table S4: Full-text exclusions and reports not retrieved; Table S5: JBI critical appraisal of included reports. The PRISMA 2020 checklist is provided as a separate Supplementary File.
